# Association Between Clinical Competencies and Mental Health Symptoms Among Frontline Medical Staff During the COVID-19 Outbreak: A Cross-Sectional Study

**DOI:** 10.3389/fpsyt.2022.760521

**Published:** 2022-04-26

**Authors:** Qing Chang, Han Su, Yang Xia, Shanyan Gao, Ming Zhang, Xiaoyu Ma, Yashu Liu, Yuhong Zhao

**Affiliations:** ^1^Department of Clinical Epidemiology, Shengjing Hospital of China Medical University, Shenyang, China; ^2^Department of Graduate Medical Education, Shengjing Hospital of China Medical University, Shenyang, China; ^3^Clinical Research Center, Shengjing Hospital of China Medical University, Shenyang, China; ^4^Office of Medical Administration and Management, Health Commission of Liaoning Province, Shenyang, China

**Keywords:** COVID-19, competency, frontline staff, medical staff, mental health

## Abstract

**Background:**

In China, mental health of frontline medical staff might be influenced by clinicians' ability to handle the outbreak of coronavirus disease 2019 (COVID-19). Few studies to-date have addressed the association between clinicians' competencies and mental health in this context. This cross-sectional study was to examine the prevalence of mental health symptoms among frontline medical staff that fought against the COVID-19 outbreak, and explore the associations between their competencies, and separate and concurrent depressive and anxiety symptoms.

**Methods:**

A total of 623 frontline medical staff was included in this study. Competencies, depressive symptoms, and anxiety symptoms were assessed using a self-reported short form of the Chinese clinical physicians' competency model, Patient Health Questionnaire-9, and Generalized Anxiety Disorder-7 questionnaire, respectively. Logistic regression models were used to evaluate the associations between one SD increase in competency scores and the prevalence of mental health problems.

**Results:**

The prevalence of depressive, anxiety, and comorbid depressive and anxiety symptoms was 40.93, 31.78, and 26.00%, respectively. Among the medical staff with higher total competency scores, the prevalence of depressive [odds ratios (ORs) = 0.67, 95% confidence intervals (CIs): 0.55–0.81], anxiety (OR = 0.68, 95% CI: 0.56–0.83), and comorbid anxiety and depressive symptoms (OR = 0.69, 95% CI: 0.55–0.83) was lower than among their lower-scoring counterparts. Subgroup analyses stratified by core competency scores revealed similar associations as the main analyses.

**Conclusion:**

The present findings highlight the association between high core competency scores and lower prevalence of depressive, anxiety, and comorbid anxiety and depressive symptoms.

## Introduction

Coronavirus disease 2019 (COVID-19), was initially reported in Wuhan, the capital of Hubei Province in China, has rapidly spread globally (1–4). The World Health Organization Emergency Committee declared COVID-19 outbreak an international public health emergency in late January 2020 (5). Wuhan soon became an epicenter of COVID-19 in China. By March 1, 2020, there have been 79,968 confirmed COVID-19 cases, including 2,873 associated deaths in China (6). The entire health care system in Wuhan, even in Hubei Province was almost paralyzed by then due to this rapidly evolving epidemic. The government of China took active measures to call for support to fight against the rapid transmission of COVID-19. Thus, over 30,000 volunteered healthcare workers from all over China were sent to Hubei Province and provided medical support to combat the COVID-19 outbreak (7, 8).

Such large-scale public health threat associated with infectious disease poses several challenges to frontline healthcare workers, who are generally medical workers treating patients with major contagious diseases. For instance, frontline medical workers are under tremendous physical and psychological pressure, higher risk of being infected, considerable work intensity, in particular, and have to cope with unknown causes and pathogens of the diseases, especially in the early stage of the COVID-19 outbreak (9, 10). The present COVID-19 epidemic has features comparable to the 2003 outbreak of severe acute respiratory syndrome (SARS-CoV), which was caused by another coronavirus, resulting in 349 deaths out of 5,327 confirmed cases in China (11). Previous studies have reported that in the initial phase of the SARS outbreak, medical staff felt vulnerable, uncertain, and concerned for their lives, which manifested with physical and psychological symptoms (12–16). It is demonstrated that frontline medical workers fighting against COVID-19 are susceptible to mental illnesses, such as depression and anxiety (10). Another study found that during the COVID-19 outbreak, frontline healthcare workers were more likely to have psychosocial issues and risk factors for developing them (17). A study carried out in Wuhan indicated that poor mental health status and sleep quality were common among frontline medical workers during the COVID-19 outbreak (18). Another survey reported that the prevalence of depression, anxiety, insomnia, and distress symptoms was 50.7, 44.7, 36.1, and 73.4%, respectively, among frontline healthcare workers in China (19). Moreover, frontline medical workers who experienced symptoms of depression were at increased risk of making errors in patient care (20). Maintaining good mental health among medical staff is essential to prevent infectious disease from spreading and ensure long-term wellbeing of staff (21, 22). Therefore, the mental health of frontline medical staff should be placed more emphasis during the outbreak of COVID-19.

Competence is a concept that refers to the ability to perform a specific activity, integrating knowledge, skills, values, and attitudes, often used to distinguish average and outstanding performance of a role (23, 24). Competence is measurable and can be developed through training. In the early stages of the COVID-19 outbreak, frontline medical workers have experienced substantial challenges with their competencies (25). Evidence shows that the core competencies are essential for healthcare workers to combat major infectious diseases outbreak (26, 27). It is demonstrated that the core competencies of nursing personnel are identified as a important factor affecting nursing effectiveness (28). Another study indicates that core competencies of nurse, including their knowledge, skills and experiences to handle unknown infectious disease, have a substantial impact on the prevention and control of the COVID-19 outbreak in China (29). In addition, it is suggested that a training program is required to improve core competencies of healthcare providers in regard to infectious disease outbreaks (30). Therefore, it is hypothesized that the high prevalence of mental disorders among frontline healthcare workers might be influenced by their competencies to handle the outbreak of COVID-19 in China. However, to the best of our knowledge, the association between competencies and mental health symptoms among frontline medical workers during the COVID-19 outbreak still remains unknown.

Therefore, the present cross-sectional study intends to examine the prevalence of mental health problems among frontline medical staff responding to the COVID-19 outbreak, and explore the associations between core competency scores and prevalence of depressive, anxiety, and comorbid anxiety and depressive symptoms in this population.

## Materials and Methods

### Participants

According to the COVID-19 pandemic isolation regulations, this cross-sectional study avoided the face to face interview and adopted online survey using an electronic questionnaire tool named “Questionnaire Star”, which was a mini-programme based on WeChat (a social media app widely used in China). Participants were able to fill out the questionnaire after scanning a quick response (QR) code by their mobile phones. Questionnaires were distributed by the directors of the medical aiding teams in WeChat group. In order to guarantee quality control of answering questionnaire, a 5-min time-frame was set in Questionnaire Star. In the pilot study, the average time of finishing the questionnaire was 5 min. Those who completed the questionnaire within 5 min would not be able to submit. Those who did not answer the questionnaire in the opening hours, or exceed the time limit for questionnaire, or have incomplete answer for the questions would be ruled out. In addition, several simple repeated questions, including age, years of service, marriage status, were set automatically in questionnaire. If the answers of the repeated questions were inconsistent, the questionnaire would be considered invalid. The entry criteria were as follows: (1) individuals aged 18 years or older; (2) frontline healthcare workers who were volunteers from different hospitals in Liaoning Province and offered medical support to cope with COVID-19 in Hubei Province; (3) without serious mental illness, such as schizophrenia. The exclusion criteria were as follows: (1) Those who did not answer the questionnaire in the opening hours; (2) Exceeding the time limit for questionnaire; (3) incomplete answer for the questions. (4) Those who failed to answer the repeated questions correct. From January 20, 2020 to February 20, 2020, which basically was the early stage of COVID-19 outbreak in China, a total of 669 frontline medical staff coping with the COVID-19 outbreak participated in the present study. Participants who failed to provide information on any of the variables were ruled out (*n* = 46). Overall, data from 623 participants were collected in the analyses. The study protocol was approved by the ethics committee of Shengjing Hospital of China Medical University, and all participants provided written informed consent to participate. The study protocol conformed to the principles of the 1975 Declaration of Helsinki.

### Assessment of Depressive Symptoms

Depressive symptoms were assessed using the Chinese version of the Patient Health Questionnaire-9 (PHQ-9), a nine-item questionnaire designed to screen for depression in primary care and other medical settings (31). Questionnaire items were scored from 0 (not at all) to 3 (nearly every day). The sum of these scores produced an overall score, ranging from 0 to 27; higher scores indicated greater depressive symptoms. The degree of depressive symptoms was classified into four categories according to the score: 0–4 points: no depression; 5–9 points: mild depression; 10–14 points: moderate depression and ≥ 15 points: severe depression (31). Those participants with an overall PHQ-9 score ≥ 5 were considered to suffer depressive symptoms (31).

### Assessment of Anxiety Symptoms

Anxiety symptoms were assessed using the Chinese version of the Generalized Anxiety Disorder-7 (GAD-7) questionnaire, which consists of 7 questions and reflects the frequency of symptoms during the preceding 2 weeks (32). Questionnaire items were scored from 0 (not at all) to 3 (nearly every day). The sum of these scores produced an overall score, ranging from 0 to 21, with higher scores indicating greater anxiety symptoms. The degree of anxiety was evaluated in accordance with the score: 0–4 points: no anxiety: 5–9 points: mild anxiety; 10–14 points: moderate anxiety and ≥ 15 points or above: severe anxiety (32). Those individuals with an overall GAD-7 score ≥ 5 were considered to suffer anxiety symptoms (32).

### Assessment of Clinicians' Competencies

Competencies among frontline medical staff were assessed using the short version of the Chinese clinical physicians' competency model, which was comprised of 12 items, classified into 8 components: clinical skills and patient care, mastery of medical knowledge, information and management, professionalism, interpersonal communication, health promotion and disease prevention, academic research, and teamwork (33, 34). In general, the items of abundant clinical experiences, skills of curing respiratory diseases, skills of treating infectious disease, skills of dealing with critical illnesses, nursing operating skills were classified into the dimension of clinical skills and patient care. The items of teamwork abilities, communication skills, good work ethic, knowledge and skills of public health, scientific research ability, accumulated medical knowledge, ability in organization and coordination and management, were categorized into the components of teamwork, interpersonal communication, professionalism, health promotion and disease prevention, academic research, mastery of medical knowledge, Information and management, respectively. Each component was weighed from 1 to 9, generating a global score from 8 to 72. Higher scores indicated greater competencies. The overall scale reliability (Cronbach's alpha) of the short version (12 items) of the Chinese Clinical physicians' competency model was 0.874 with dimensions ranged from 0.737 to 0.892. The test-retest reliability examined after 2 weeks was 0.905. The validity of the short version of the Chinese Clinical physicians' competency model was also examined. The Spearman correlation coefficient with the full version (103 items) was 0.833. Correlation coefficients at the item level between the two versions of scale ranged from 0.810 to 0.975.

### Assessment and Definitions of Confounding Factors

All confounding factors (age, gender, profession, department, marital status, relative infected by COVID-19, working location, household income, years of service, working time after the COVID-19 outbreak, and sleeping time after COVID-19) were collected using a questionnaire. For analyses, profession was classified as “physician”, “nurse”, or “public health practitioner”; department was classified as “intensive care unit”, “general ward”, or “other”; household income was classified as “≥1,00,000 Yuan/year” or below; marital status was classified as “currently married” or “currently unmarried”; working location was classified as “Hubei province” or “other”.

### Statistical Analysis

Participant characteristics were reported stratified by depressive or anxiety symptoms status. Continuous variables were presented as least-square means and 95% confidence intervals (CIs); categorical variables were presented as counts and percentages. Logistic regression models were used to estimate the associations between one standard deviation increase in clinicians' competency scores, and the prevalence of depressive and anxiety symptoms. Odds ratios (ORs) and 95% CIs were calculated. The crude model was used to calculate the crude OR (95% CI) without any adjustments. Model 1 was adjusted for age and sex. Model 2 was further adjusted for profession, department, marital status, relative infected by COVID-19, working location, household income, years of service, working time after COVID-19, and sleeping time after COVID-19. Moreover, in order to increase analytic rigor, we also conducted the Benjamini-Hochberg procedure to calculate adjusted *P*-values. All analyses were performed using the Statistical Analysis System 9.3 edition for Windows (SAS Institute Inc., Cary, NC, USA). All *P*-values were two-tailed, and *P*-values < 0.05 were considered statistically significant.

## Results

### Participant Characteristics

Among 623 participants, the prevalence of depressive, anxiety, and comorbid depressive and anxiety symptoms was 40.93, 31.78, and 26.00%, respectively. The mean age was 33.93 (95% CI: 33.74–34.49) years.

Participant characteristics are presented in Tables 1, 2, divided by depressive and anxiety symptoms status, respectively. Participants with depressive or anxiety symptoms had lower clinicians' competency scores. Participants with depressive symptoms tended to be younger (*P* = 0.01), not married (*P* = 0.03), and have a shorter length of service (*P* = 0.01). Moreover, participants with anxiety symptoms were more likely to be men (*P* = 0.02), younger (*P* < 0.01), working at departments other than intensive care or general wards (*P* < 0.001), based in provinces other than Hubei (*P* < 0.001), with a shorter length of service (*P* < 0.01), and a higher likelihood of having a relative infected by COVID-19 (*P* = 0.02).

**Table 1:** Participant characteristics, stratified by the presence of depressive symptoms^a^.

**Characteristic**	**Depressive symptoms status**		***P*-value^**b**^**	***P adj*.**
	**No**	**Yes**		
PHQ-9 scores (≥5, %)	368 (59.07)	255 (40.93)		
PHQ-9 scores (mean value)	1.53 (1.26, 1.81)^c^	8.83 (8.50, 9.16)		
Total competencies score	61.75 (60.64, 62.87)	57.48 (56.14, 58.82)	<0.001	<0.01
Information and management	7.77 (7.60, 7.93)	7.27 (7.08, 7.47)	<0.001	<0.01
Professionalism	8.40 (8.25, 8.54)	7.92 (7.47, 8.09)	<0.001	<0.01
Clinical skills and patient care	7.21 (7.03, 7.40)	6.59 (6.37, 6.81)	<0.001	<0.01
Interpersonal communication	8.13 (7.97, 8.28)	7.53 (7.35, 7.72)	<0.001	<0.01
Health promotion and disease prevention	7.71 (7.54, 7.89)	7.18 (6.97, 7.39)	<0.001	<0.01
Mastery of medical knowledge	7.99 (7.83, 8.15)	7.51 (7.32, 7.70)	<0.001	<0.01
Academic research	6.23 (6.01, 6.46)	5.73 (5.46, 6.01)	<0.01	<0.05
Teamwork	8.31 (8.17, 8.46)	7.75 (7.57, 7.93)	<0.001	<0.01
Sex (male, %)	32.88	38.04	0.18	0.25
Age (years)	34.54 (33.81, 35.26)	33.06 (32.19, 33.92)	0.01	<0.05
Years of service	11.18 (10.51, 11.86)	9.80 (8.98, 10.61)	0.01	<0.05
Married (yes, %)	79.62	72.16	0.03	0.06
Household income (≥100,000 Yuan/year, %)	54.62	50.20	0.28	0.35
Working time after COVID-19 (hours/day)	8.45 (8.17, 8.72)	8.13 (7.79, 8.46)	0.15	0.23
Sleeping time after COVID-19 (hours/day)	6.82 (6.67, 6.98)	6.67 (6.49, 6.86)	0.22	0.29
Profession				
Physician	28.80	27.06	0.63	0.69
Nurse	60.60	61.96	0.73	0.76
Public health practitioner	10.60	10.98	0.88	0.88
Department				
Intensive care unit	38.86	36.86	0.61	0.7
General ward	34.24	30.59	0.34	0.41
Other	26.90	32.55	0.13	0.21
Relative infected by COVID-19	5.71	9.80	0.05	0.09
Working location (Hubei province, %)	75.54	69.02	0.07	0.12

a *PHQ-9, Patient Health Questionnaire-9; COVID-19, Coronavirus disease 2019*.

b *Analysis of variance or chi-square test*.

c *Least square mean (95% confidence interval) (all reported values)*.

**Table 2:** Participant characteristics, stratified by the presence of anxiety symptoms^a^.

**Characteristic**	**Anxiety symptoms status**		***P*-value^**b**^**	***P adj*.**
	**No**	**Yes**		
GAD-7 scores (≥5, %)	425 (68.22)	198 (31.78)		
GAD-7 scores (mean value)	1.33 (1.13, 1.53)^c^	8.04 (7.75, 8.33)		
Total competencies score	61.57 (60.54, 62.61)	56.64 (55.13, 58.16)	<0.001	<0.01
Information and management	7.78 (7.63, 7.93)	7.11 (6.88, 7.33)	<0.001	<0.01
Professionalism	8.43 (8.30, 8.56)	7.71 (7.52, 7.90)	<0.001	<0.01
Clinical skills and patient care	7.16 (6.99, 7.33)	6.52 (6.27, 6.77)	<0.001	<0.01
Interpersonal communication	8.09 (7.95, 8.24)	7.43 (7.22, 7.64)	<0.001	<0.01
Health promotion and disease prevention	7.69 (7.53, 7.85)	7.09 (6.85, 7.32)	<0.001	<0.01
Mastery of medical knowledge	8.00 (7.85, 8.14)	7.35 (7.13, 7.56)	<0.001	<0.01
Academic research	6.11 (5.90, 6.32)	5.86 (5.55, 6.17)	0.19	0.28
Teamwork	8.31 (8.17, 8.45)	7.59 (7.39, 7.79)	<0.001	<0.01
Sex (male, %)	32.00	41.41	0.02	<0.05
Age (years)	34.48 (33.81, 35.15)	32.75 (31.77, 33.73)	<0.01	0.02
Years of service	11.19 (10.56, 11.82)	9.38 (8.46, 10.30)	<0.01	0.02
Married (yes, %)	77.88	73.74	0.26	0.35
Household income (≥100,000 Yuan/year, %)	54.59	48.99	0.19	0.28
Working time after COVID-19 (hours/day)	8.26 (8.00, 8.52)	8.44 (8.06, 8.82)	0.43	0.52
Sleeping time after COVID-19 (hours/day)	6.76 (6.62, 6.91)	6.76 (6.55, 6.97)	0.96	0.96
Profession				
Physician	28.47	27.27	0.76	0.79
Nurse	61.65	60.10	0.71	0.77
Public health practitioner	9.88	12.63	0.30	0.38
Department				
Intensive care unit	42.12	29.29	<0.01	0.02
General ward	33.41	31.31	0.60	0.69
Other	24.47	39.39	<0.001	<0.01
Relative infected by COVID-19	5.65	11.11	0.02	<0.05
Working location (Hubei province, %)	77.18	63.64	<0.001	<0.01

a *GAD, Generalized Anxiety Disorder-7; COVID-19, Coronavirus disease 2019*.

b *Analysis of variance or chi-square test*.

c *Least square mean (95% confidence interval) (all reported values)*.

### Clinicians' Competencies and Depressive Symptoms

Associations between clinicians' competency scores and prevalence of depressive symptoms are presented in Table 3. Among participants with higher total competency scores, the prevalence of depressive symptoms was lower. The multivariate-adjusted OR (95% CI) for depressive symptoms per one standard deviation increase in total scores of competencies was 0.67 (0.55–0.81). Furthermore, subgroup analyses stratified by eight core competency categories revealed similar associations as the total score. After corrected for multiple comparisons, the results were not changed.

**Table 3:** Associations between competencies, anxiety, and depressive symptoms among frontline medical staff during the COVID-19 outbreak.

				**Adjusted**		**Adjusted**			
	**Crude model**	***P***	***P*_**adj.**_**	**model 1^**a**^**	***P***	***P*_**adj.**_**	**model 2^**b**^**	***P***	***P*_**adj.**_**
**Depressive symptoms**									
Total competencies score	0.67 (0.57, 0.80)^c^	<0.0001	0.0003	0.70 (0.58, 0.83)	<0.0001	0.0005	0.67 (0.55, 0.81)	<0.0001	0.0004
Information and management	0.74 (0.63, 0.87)	0.0003	0.0003	0.77 (0.65, 0.90)	0.0017	0.0019	0.76 (0.63, 0.90)	0.0021	0.0025
Professionalism	0.71 (0.59, 0.83)	<0.0001	0.0003	0.74 (0.62, 0.88)	0.0007	0.0011	0.71 (0.58, 0.85)	0.0004	0.0007
Clinical skills and patient care	0.71 (0.60, 0.83)	<0.0001	0.0003	0.73 (0.62, 0.86)	0.0002	0.0005	0.69 (0.57, 0.83)	<0.0001	0.0004
Interpersonal communication	0.67 (0.56, 0.79)	<0.0001	0.0003	0.70 (0.58, 0.83)	<0.0001	0.0005	0.68 (0.56, 0.82)	<0.0001	0.0004
Health promotion and disease prevention	0.73 (0.62, 0.86)	0.0001	0.0002	0.75 (0.63, 0.88)	0.0006	0.0011	0.74 (0.62, 0.88)	0.0008	0.0012
Mastery of medical knowledge	0.73 (0.62, 0.86)	0.0002	0.0003	0.76 (0.64, 0.90)	0.0012	0.0015	0.76 (0.63, 0.90)	0.0021	0.0025
Academic research	0.80 (0.68, 0.94)	0.0060	0.0060	0.81 (0.69, 0.96)	0.0130	0.0130	0.82 (0.69, 0.97)	0.0179	0.0179
Teamwork	0.67 (0.56, 0.80)	<0.0001	0.0003	0.70 (0.58, 0.83)	<0.0001	0.0005	0.67 (0.55, 0.81)	<0.0001	0.0004
**Anxiety symptoms**									
Total competencies score	0.65 (0.55, 0.77)	<0.0001	0.0002	0.68 (0.57, 0.81)	<0.0001	0.0003	0.68 (0.56, 0.83)	0.0001	0.0003
Information and management	0.67 (0.57, 0.79)	<0.0001	0.0002	0.70 (0.59, 0.83)	<0.0001	0.0003	0.70 (0.58, 0.84)	0.0001	0.0003
Professionalism	0.61 (0.51, 0.72)	<0.0001	0.0002	0.64 (0.53, 0.76)	<0.0001	0.0003	0.63 (0.52, 0.77)	<0.0001	0.0006
Clinical skills and patient care	0.71 (0.60, 0.84)	<0.0001	0.0002	0.74 (0.62, 0.87)	0.0004	0.0005	0.74 (0.61, 0.89)	0.0019	0.0023
Interpersonal communication	0.66 (0.55, 0.78)	<0.0001	0.0002	0.69 (0.58, 0.82)	<0.0001	0.0003	0.70 (0.58, 0.85)	0.0003	0.0005
Health promotion and disease prevention	0.71 (0.60, 0.84)	<0.0001	0.0002	0.74 (0.62, 0.88)	0.0005	0.0006	0.75 (0.62, 0.90)	0.0019	0.0023
Master of medical knowledge	0.67 (0.57, 0.79)	<0.0001	0.0002	0.70 (0.59, 0.83)	<0.0001	0.0003	0.72 (0.59, 0.86)	0.0004	0.0006
Academic research	0.90 (0.76, 1.06)	0.1926	0.1926	0.92 (0.78, 1.09)	0.3335	0.3335	0.92 (0.77, 1.10)	0.3668	0.3668
Teamwork	0.62 (0.52, 0.74)	<0.0001	0.0002	0.66 (0.55, 0.78)	<0.0001	0.0003	0.66 (0.54, 0.80)	<0.0001	0.0006

a *Adjusted for age and gender*.

b *Adjusted for age, gender, profession, department, marriage status, relative infected by COVID-19, working location, household income, years of service, working time after COVID-19, and sleeping time the COVID-19outbreak*.

c *Odds ratio (95% confidence interval) per one standard deviation increase of scores of clinicians' competencies (all reported values)*.

### Clinicians' Competencies and Anxiety Symptoms

As shown in Table 3, total clinicians' competency scores were negatively associated with the prevalence of anxiety symptoms. After adjustments for confounding factors, the OR (95% CI) for anxiety symptoms per one standard deviation increase in the total competency score was 0.68 (0.56–0.83). Moreover, scores on seven core competency categories (all except academic research) were negatively associated with the prevalence of anxiety symptoms. After corrected for multiple comparisons, the results were not changed.

### Clinicians' Competencies and Comorbid Anxiety and Depressive Symptoms

We examined associations between clinicians' competency scores and comorbid anxiety and depressive symptoms (Table 4). Among participants with higher total competency scores, the prevalence of comorbid anxiety and depressive symptoms was lower than among their counterparts. The OR (95% CI) for comorbid anxiety and depressive symptoms per one standard deviation increase in total clinicians' competency score was 0.68 (0.55–0.83). Seven core competency categories (all except academic research) were negatively associated with the prevalence of comorbid anxiety and depressive symptoms. After corrected for multiple comparisons, the results were not changed.

**Table 4:** Associations between competencies, and comorbid anxiety and depressive symptoms among frontline medical staff during the COVID-19 outbreak.

				**Adjusted**			**Adjusted**		
	**Crude model**	***P***	***P*_**adj.**_**	**model 1^**a**^**	***P***	***P*_**adj.**_**	**model 2^**b**^**	***P***	***P*_**adj.**_**
**Comorbid anxiety and depressive symptoms**									
Total competencies score	0.64 (0.53, 0.76)^c^	<0.0001	0.0002	0.68 (0.56, 0.81)	<0.0001	0.0003	0.68 (0.55, 0.83)	0.0001	0.0003
Information and management	0.69 (0.58, 0.82)	<0.0001	0.0002	0.73 (0.61, 0.88)	0.0006	0.0009	0.73 (0.61, 0.89)	0.0014	0.0021
Professionalism	0.58 (0.49, 0.69)	<0.0001	0.0002	0.62 (0.51, 0.74)	<0.0001	0.0003	0.62 (0.50, 0.75)	<0.0001	0.0006
Clinical skills and patient care	0.72 (0.60, 0.85)	0.0002	0.0002	0.75 (0.63, 0.90)	0.0015	0.0017	0.76 (0.62, 0.92)	0.0061	0.0069
Interpersonal communication	0.64 (0.54, 0.76)	<0.0001	0.0002	0.68 (0.57, 0.81)	<0.0001	0.0003	0.70 (0.57, 0.84)	0.0003	0.0007
Health promotion and disease prevention	0.70 (0.59, 0.84)	<0.0001	0.0002	0.74 (0.62, 0.88)	0.0008	0.0010	0.75 (0.62, 0.91)	0.0031	0.0040
Master of medical knowledge	0.66 (0.55, 0.78)	<0.0001	0.0002	0.69 (0.58, 0.83)	<0.0001	0.0003	0.71 (0.58, 0.86)	0.0004	0.0007
Academic research	0.85 (0.71, 1.02)	0.0736	0.0736	0.88 (0.73, 1.05)	0.1584	0.1584	0.87 (0.72, 1.05)	0.1455	0.1455
Teamwork	0.60 (0.50, 0.72)	<0.0001	0.0002	0.64 (0.53, 0.77)	<0.0001	0.0003	0.64 (0.52, 0.78)	<0.0001	0.0006

a *Adjusted for age and gender*.

b *Adjusted for age, gender, profession, department, marriage status, relative infected by COVID-19, working location, household income, years of service, working time after COVID-19, and sleeping time after the COVID-19 outbreak*.

c *Odds ratio (95% confidence interval) per one standard deviation increase of scores of clinicians' competencies (all reported values)*.

## Discussion

To the best of our knowledge, the present study is the first study to explore the associations between competencies and mental health symptoms among frontline healthcare workers fighting against the COVID-19 outbreak. Mental health problems, including depressive, anxiety, and comorbid depressive and anxiety symptoms are highly prevalent in frontline healthcare workers. Our findings suggest that higher competency scores are associated with lower prevalence of depressive, anxiety, and comorbid depressive and anxiety symptoms. Subgroup analyses stratified by different domains of competencies yielded results similar to the results of the main analyses.

It is well established that the outbreak of severe infectious diseases may exert adverse psychological impact on ordinary people and healthcare staff. A number of studies have confirmed that medical staff suffered mental illnesses during the outbreak of SARS in 2003 (14–16). The present findings suggest that frontline healthcare workers with depressive and anxiety symptoms tend to be younger and have a shorter length of service. The risk of depressive and anxiety symptoms appears to be influenced by frontline healthcare workers' age and length of service (35, 36). Possible factors that account for these findings are age- and service duration-related decrease in emotional responsiveness, and increase in emotional control and psychological resilience.

In the present study, the prevalence of depressive, anxiety, and comorbid depressive and anxiety symptoms among Chinese frontline medical workers fighting against the COVID-19 outbreak was 40.93, 31.78, and 26.00%, respectively, which was higher than reported peacetime estimates as well as estimates from the initial phase of the SARS epidemic (37–41). A previous study conducted in southern China has reported that 28.13% of physicians had depressive symptoms, 25.67% had anxiety symptoms, and 19.01% had comorbid depressive and anxiety symptoms (38). Another study has explored the impact of the SARS epidemic on healthcare workers in Taiwan, reporting the prevalence of depressive symptoms at 17.3% during the SARS epidemic (41). In a recent multi-center survey, high prevalence of depressive (50.7%) and anxiety (44.7%) symptoms of frontline medical workers has been reported during the COVID-19 outbreak in China (21). Mental health problems among medical staff might hinder their professional performance and affect the quality of response to COVID-19. Concurrently, deterioration in medical workers' wellbeing is likely to negatively affect on patients and professionals' overall health. Protecting mental health of medical staff is crucial for epidemic control and maintaining staff wellbeing.

In recent years, clinical core competencies, including clinical skills and patient care, mastery of medical knowledge, health promotion and disease prevention, information and management, professionalism, interpersonal communication, academic research, and teamwork have played a key role in defining medical staff's ability worldwide (42–45). High competency scores indicate good clinical performances.

Moreover, the present study found that lower competency scores have been associated with increased prevalence of anxiety, depressive, and comorbid anxiety and depressive symptoms. Competency scores were negatively associated with the prevalence of mental health problems among frontline medical staff. These findings suggest that improving core competencies among frontline medical workers coping with the epidemic might help contain the spread of COVID-19. To control the epidemic, health authorities would ensure that frontline workers are competent and equipped with up-to-date knowledge and information.

To the best of our knowledge, the present study is the first to use a competency-based survey to investigate the associations between core competency scores and the prevalence of mental health problems among frontline medical workers. The present findings highlight the importance of clinicians' competencies in maintaining mental health. Suitable training should be provided to frontline medical workers. Nevertheless, this study has several limitations, which should be considered when interpreting its findings. Firstly, the cross-sectional design of the present study limits discussions about causality and generalizability of the findings. For example, participants with worse mental health may assess their competencies more negatively. Secondly, the use of an online survey might have resulted in a biased participant sample. However, given the high transmission rate of the virus, which has restricted the opportunities to conduct face-to-face surveys, WeChat-based survey programme Questionnaire Star has been widely implemented in China (21). Thirdly, unmeasured confounding factors might have affected the observed findings. Fourthly, in the present study, considering the heavy work for the frontline medical staff in the early stage of COVID-19 outbreak, we used a short version of the Chinese Clinical physicians' competency model to evaluate the competencies. Even though the short version has been validated in medical staff (including doctor, nurse and other types of medical staff), comparing to the full version, it cannot reflect all the characteristics of frontline medical staff's competencies. Fifthly, due to the COVID-19 pandemic isolation regulations and the risk of virus transmission, a self-reported clinical competency scale was used in the present study. Even though the scale showed decent reliability and validity, due to the nature of self-reported questionary, recall bias and reporting bias exist and the associations between clinical competency and depression may be overestimated. Future cohort studies with objective assessments of clinical competencies are needed to classify the results.

In conclusion, mental health problems associated with core competencies are highly prevalent among frontline medical workers combating the COVID-19 outbreak. Protecting mental health of medical workers is of great importance for epidemic control. Our study has highlighted the importance of clinicians' core competencies in maintaining staff well-being during an epidemic. Providing additional training to frontline medical staff might help prevent the onset of mental health problems and make efforts to contain COVID-19. To address the COVID-19 epidemic, health authorities would ensure competent staff, equipped with up-to-date information are volunteered to combat in the frontline.

## Data Availability Statement

The original contributions presented in the study are included in the article/supplementary material, further inquiries can be directed to the corresponding author.

## Ethics Statement

The studies involving human participants were reviewed and approved by the Ethics Committee of Shengjing Hospital of China Medical University. The patients/participants provided their written informed consent to participate in this study.

## Author Contributions

QC and YZ designed the study and formulated the clinical question. YZ had full access to all data in the study and is responsible for data integrity and the accuracy of data analysis. All authors collected, managed, analyzed the data, prepared, reviewed, revised, and read and approved the final manuscript.

## Funding

This work was supported by the National Key R&D Program of China (No. 2017YFC0907404 to YZ), Liaoning Revitalization Talents Program (No. XLYC1802095 to YZ), the Science and Technology Project of Liaoning Province (No. 2019JH6/10400002 to YZ), the Young Faculty support program of China Medical University (No. QGRA2018002 to QC), the Natural Science Foundation of Liaoning Province of China (No. 2020-MS-184 to QC), the 345 Talent Project of Shengjing Hospital of China Medical University (No. M0715 to QC), and the Young Talents of Education Ministry of Liaoning Province (No. QN2019011 to HS).

## Conflict of Interest

The authors declare that the research was conducted in the absence of any commercial or financial relationships that could be construed as a potential conflict of interest.

## Publisher's Note

All claims expressed in this article are solely those of the authors and do not necessarily represent those of their affiliated organizations, or those of the publisher, the editors and the reviewers. Any product that may be evaluated in this article, or claim that may be made by its manufacturer, is not guaranteed or endorsed by the publisher.
